# Effect of Scalp Nerve Block with Ropivacaine on Postoperative Pain in Patients Undergoing Craniotomy: A Randomized, Double Blinded Study

**DOI:** 10.1038/s41598-020-59370-z

**Published:** 2020-02-13

**Authors:** Yaoxin Yang, Mengchan Ou, Hongyu Zhou, Lingcan Tan, Yajiao Hu, Yu Li, Tao Zhu

**Affiliations:** 1 Department of Anesthesiology, West China Hospital of Sichuan University, Chengdu, 610041 Sichuan P.R. China; 2 Laboratory of Anesthesia & Critical Care, Translational Neuroscience Center, West China Hospital of Sichuan University, Chengdu, 610041 Sichuan P.R. China

**Keywords:** Sensory processing, Neurological disorders

## Abstract

Scalp nerve block with ropivacaine has been shown to provide perioperative analgesia. However, the best concentration of ropivacaine is still unknown for optimal analgesic effects. We performed a prospective study to evaluate the effects of scalp nerve block with varied concentration of ropivacaine on postoperative pain and intraoperative hemodynamic variables in patients undergoing craniotomy under general anesthesia. Eighty-five patients were randomly assigned to receive scalp block with either 0.2% ropivacaine, 0.33% ropivacaine, 0.5% ropivacaine, or normal saline. Intraoperative hemodynamics and post-operative pain scores at 2, 4, 6, 24 hours postoperatively were recorded. We found that scalp blockage with 0.2% and 0.33% ropivacaine provided adequate postoperative pain relief up to 2 h, while administration of 0.5% ropivacaine had a longer duration of action (up to 4 hour after craniotomy). Scalp nerve block with varied concentration of ropivacaine blunted the increase of mean arterial pressure in response to noxious stimuli during incision, drilling, and sawing skull bone. 0.2% and 0.5% ropivacaine decreased heart rate response to incision and drilling. We concluded that scalp block using 0.5% ropivacaine obtain preferable postoperative analgesia compared to lower concentrations. And scalp block with ropivacaine also reduced hemodynamic fluctuations in craniotomy operations.

## Introduction

About 10% to 20% patients undergoing craniotomy suffered severe pain and more than 30% experienced moderate pain as per *Guilfoyle et al*.^1^. These experiences with pain may disturb patient sleep patterns and prolong hospital stays^2^. Abrupt increases in heart rate (HR) and blood pressure (BP) resulting from dramatic stimuli like incisions, drilling, and screwing cause potential morbidities and mortalities due to elevation of intracranial pressure (ICP) in patients^3,4^. Generally, opioids are used for relieving hemodynamic fluctuations and reducing postoperative pain, however, it may delay recovery time, contribute to extreme sedation, and interfere with postoperative neurological examinations. In addition, adverse effects of opioids such as nausea and vomiting, and respiratory depression may result in a rise of ICP or mask the signs of increased ICP. Since there is such an emphasis on controlling the adverse effects of opioid administration, postoperative pain after craniotomy is frequently uncontrolled^1^. Easing hemodynamic perturbation and relieving postoperative pain are important concerns of neuroanesthesiologists and are also necessary components of the Enhanced Recovery After Surgery (ERAS). With advances in modern anesthesia come the development of short-acting analgesics, mainly remifentanil, transition analgesics, and conjunction analgesics that can be used instead of opioids to treat postoperative pain^5^.

Scalp never block (SNB), the blockage of nerves that innervate the involved region of the scalp about surgery^6^, was developed due to its potential benefits for effective regional anesthesia administration^7^, which promotes development of precise neurosurgery, such as functional and micro neurosurgery. Many researchers demonstrated that SNB attenuate autonomic responses and provided sufficient postoperative analgesia^1,3,5,8,9^.

Ropivacaine is the drug of choice for administration of a local nerve block due to its longer duration of action compared with lidocaine and less cardiac and central nervous system toxicity than bupivacaine^10,11^. Although many researches pointed out SNB was effective and convenient, plasma concentration of local anesthetics increased rapidly after injection, unlike other neural blockades^12–15^. *Audu et al*.^14^ suggested that the peak plasm concentration level of either 0.35% or 0.5% ropivacaine occurred within 13 minutes of the commencement of scalp infiltration. Although zero patients had any signs of local anesthetic toxicity, a few patients reached excessive peak levels of ropivacaine, which have been previously reported to potentially develop into CNS toxicity symptoms in healthy volunteers^10^. *Archer DP et al*.^16^ reported seven patients with signs compatible with local anesthetic toxicity shortly after local agent injection during craniotomy.

We postulate that if a low concentration of ropivacaine for pre-incisional SNB achieved a similarly analgesic efficacy by reducing the incidence of absorption-related toxicity of local anesthetics, the safety of SNB would be enhanced. Accordingly, this study aimed to evaluate the effect of SNB using different concentrations of ropivacaine (0.2%, 0.33% and 0.5%) on postoperative pain and hemodynamic responses in patients undergoing craniotomy under general anesthesia.

## Results

**Figure 1:**
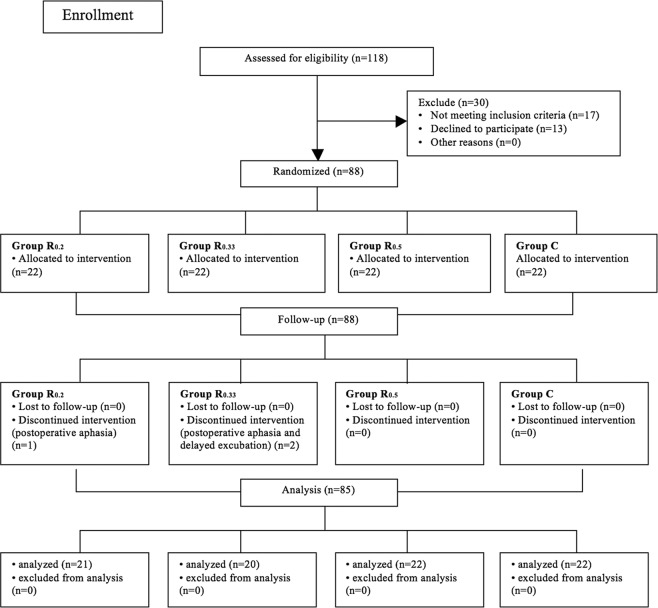
Diagram for Patients enrollment and follow-up.

One hundred and eighteen patients were screened for this prospective, randomized, doubled-blind, placebo-controlled study. Of these, eighty-eight patients were recruited and randomly assigned to received different interventions (Fig. 1). Among them, three patients were excluded before completing the protocol due to surgical complication: one patient appeared with delayed extubation, while two cases presented with postoperative aphasia. In total, eighty-five patients completed this study.

Multiple comparison results indicated that no significant differences were detected among groups in demographic characteristics of patients and operative variables including age, sex, body mass index, ASA status, the duration of operation and total dose of remifentanil (Table 1).

**Table 1:** Patients characteristics and operative variables.

Characteristic	Group R_0.2_(n = 21)	Group R_0.33_(n = 20)	Group R_0.5_(n = 22)	Group C(n = 22)
Sex(female/male)^#^	10/11	8/12	11/11	15/7
Age(year)*	43.14 ± 12.18	42.55 ± 11.98	46.41 ± 11.26	44.14 ± 13.28
Body Mass Index*	22.95 ± 2.53	21.47 ± 2.60	21.54 ± 1.89	22.31 ± 2.16
ASA(I/II)^#^	8/13	9/11	9/13	9/13
Duration of operation(hours)*	4.39 ± 0.71	4.38 ± 0.57	4.27 ± 0.69	4.17 ± 0.60
Total dose of remifentanil(mg)*	1.65 ± 0.32	1.53 ± 0.31	1.47 ± 0.31	1.49 ± 0.26

^*^Values are expressed as means ± SD. ^#^Data are presented as total number of patients (n).

ASA indicates American Society of Anaesthesiologists.

**Figure 2:**
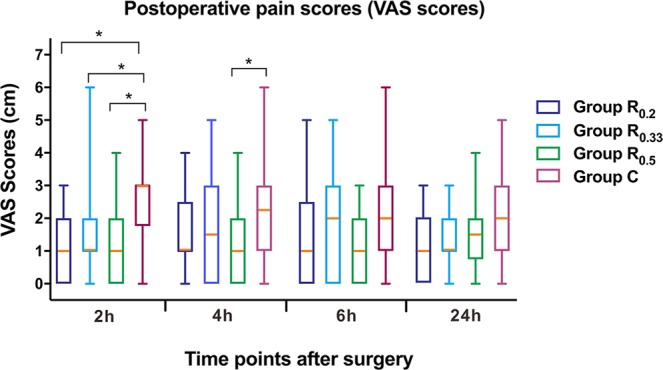
Comparison of postoperative visual analogue scale (VAS) scores among four groups. Group R_0.2_ = 0.2% ropivacaine, Group R_0.33_ = 0.33% ropivacaine, Group R_0.5_ = 0.5% ropivacaine, Group C = normal saline. *P < 0.05.

Reported pain at postoperative 2 hours in the groups R_0.2_, R_0.33_ and R_0.5_ exhibited significantly lower VAS scores than group C (P = 0.012, 0.021 and 0.012, respectively), while only group R_0.5_ have a significantly lower VAS score 4 hours post operation (P = 0.023) (Fig. 2). Furthermore, stratification analysis of the surgerical duration found when the length of neurosurgical procedures were more than four hours, groups R_0.2_, R_0.33_ and R_0.5_ exhibit significantly lower VAS scores than group C at postoperative 2 hours (Table 2). However, no significant difference among groups when duration less than 4 h. However, there was no significant difference among the group R_0.2_, R_0.33_ and R_0.5_ in VAS scores.

**Table 2:** Stratification analysis of the duration of surgery.

	Duration >4 h (n = 52)				Duration ≤4 h (n = 33)			
	VAS 2 h	VAS 4 h	VAS 6 h	VAS 24 h	VAS 2 h	VAS 4 h	VAS 6 h	VAS 24 h
Group R_0.2_	1 (0, 2.25)	1 (1,2.25)	1 (0.75, 2)	1.5 (0, 2)	1 (1, 2)	1 (0, 4)	1 (0, 4)	1 (0, 2)
Group R_0.33_	1 (1, 2)	2 (0.75, 3)	2 (0, 3)	1 (0.75, 2)	1 (0, 2.25)	0.5 (0.5, 3.5)	1.5 (0.75, 4.25)	2 (0.785, 2.25)
Group R_0.5_	1 (0, 2.5)	1 (0, 2.5)	1 (1, 2)	2 (0.5, 2)	1 (0.5, 2)	1 (0.5, 1.5)	1 (0, 1.75)	1 (0.5, 2.5)
Group C	3 (2, 3)	2.5 (2, 3)	2 (2, 3)	2 (1, 3)	3 (1, 4)	2 (1, 5)	1 (1,3)	1 (0, 3)
P Value	0.035	0.23	0.151	0.264	0.156	0.298	0.691	0.858

Data are presented as median and quartiles.

**Figure 3:**
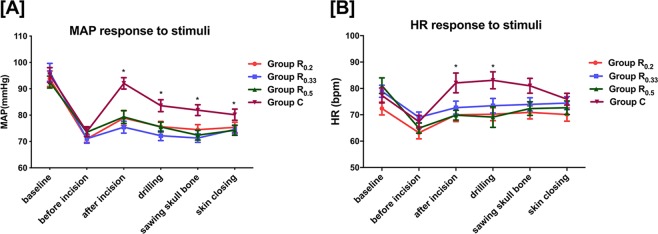
Changes in mean artery pressure (MAP) (mmHg) (**A**) and heart rate (HR) (beat per minute) (**B**) of four groups at time points of baseline, before and after skin incision, drilling, sawing skull bone and skin closure. *P < 0.05.

The MAP and HR in the four groups were shown in Fig. 3(A,B). Patients in group R_0.2_, R_0.33_ and R_0.5_ reported significantly less MAP than did those in group C at the time of skin incision, drilling, sawing skull bone, and skin closure. Additionally intraoperative HR was significantly lower in Group R_0.2_ and R_0.5_ compared to group C at incision drilling and sawing. GLMM results showed that at time of baseline, before and after incision, MAP had statistically significant changes over time (Supplementary Table 2). Similarly, at time point of baseline, before incision, and drilling, HR also had significant difference over time (Supplementary Table 5). But both in MAP and HR, the covariance of time was not statistically significant, indicating that the trend of MAP and HR changes in patients was similar (Supplementary Tables 3, 6). There was no significant difference over time in VAS scores (Supplementary Tables 7–9).

**Figure 4:**
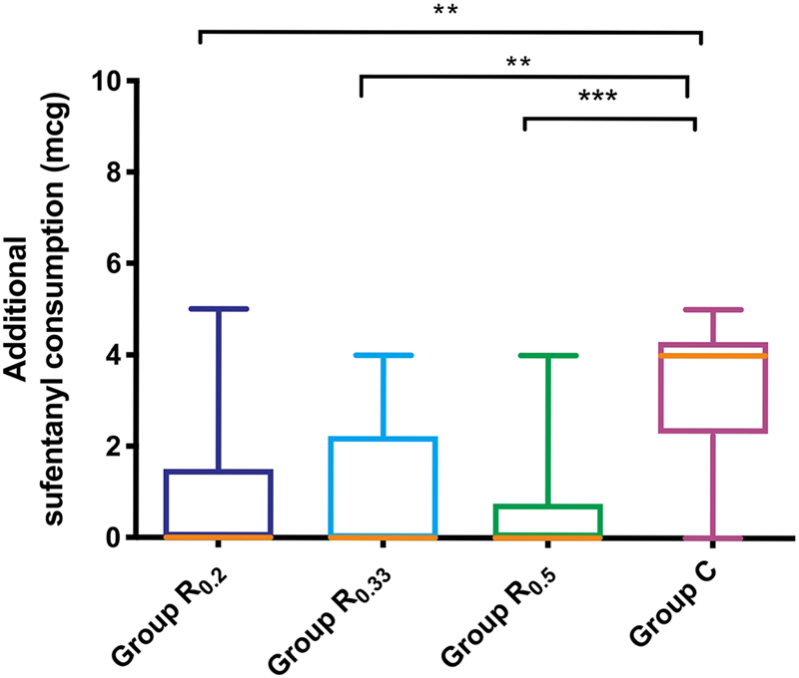
Comparison of intraoperative additional sufentanyl requirements during surgery among four groups. Group R_0.2_ = 0.2% ropivacaine, Group R_0.33_ = 0.33% ropivacaine, Group R_0.5_ = 0.5% ropivacaine, Group C = normal saline. **P = 0.001, ***P < 0.001.

**Figure 5:**
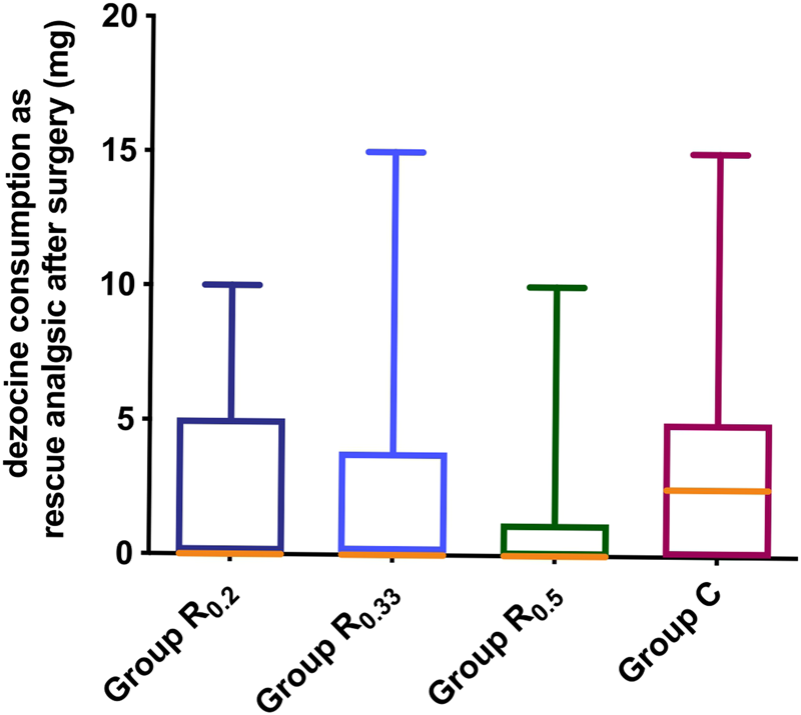
Comparison of dezocine consumption as rescue analgesic after surgery among four groups. Group R_0.2_ = 0.2% ropivacaine, Group R_0.33_ = 0.33% ropivacaine, Group R_0.5_ = 0.5% ropivacaine, Group C = normal saline. (P = 0.214).

**Figure 6:**
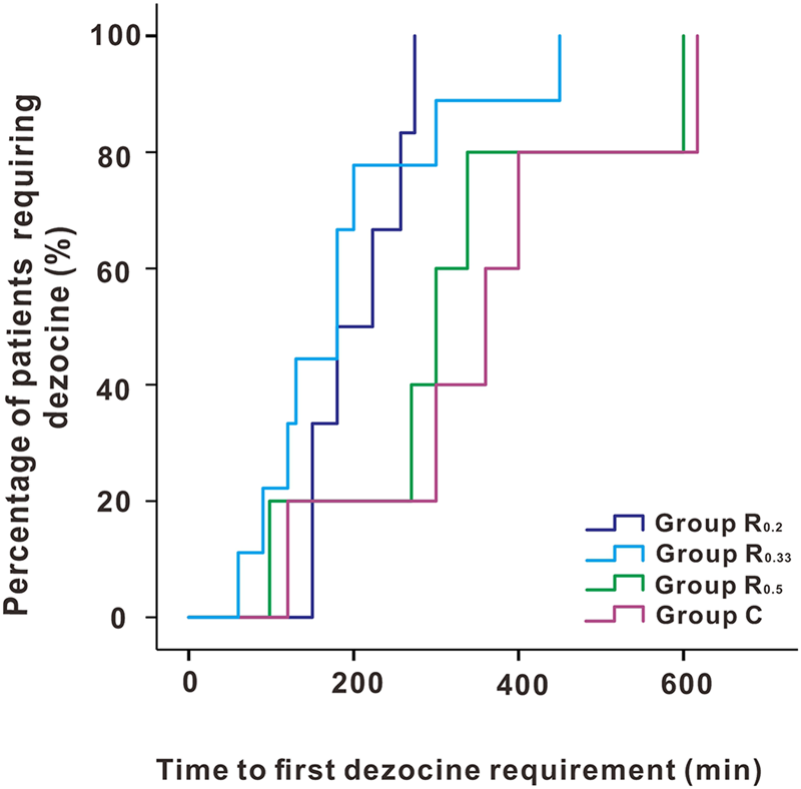
Kaplan-Meier curve depicting time to first dezocine requirement after surgery among four groups. Group R_0.2_ = 0.2% ropivacaine, Group R_0.33_ = 0.33% ropivacaine, Group R_0.5_ = 0.5% ropivacaine, Group C = normal saline. (P = 0.091).

Figure 4 displayed additional intraoperative sufentanyl requirements which were significantly higher in group C compared to group R_0.2_, group R_0.33_ and group R_0.5_, while the latter three groups were not significantly different. The dezocine consumption as rescue analgesic and time to first requirement of dezocine after surgery were displayed in Figs. 5 and 6, which exhibited no significant differences among groups. Table 3 showed there was no statistical difference in the incidence of PONV among groups. In our study, post-operative scalp infection or hematoma, and local or general complications were absent in patients during the study period.

**Table 3:** The incidence of nausea and vomiting after surgery.

Time interval	Group R_0.2_(n = 21)	Group R_0.33_(n = 20)	Group R_0.5_(n = 22)	Group C(n = 22)	P value
2 h	2/21	0/20	1/22	1/22	0.557
4 h	0/21	1/20	0/22	2/22	0.296
6 h	1/21	3/20	3/22	3/22	0.719
24 h	1/21	1/20	3/22	1/22	0.581

Data are presented as total number of patients (n).

## Discussion

The results of our study showed that SNB with 0.2%, 0.33%, 0.5% ropivacaine relieved postoperative pain for up to 2 hours after elective craniotomy under general anesthesia. Meanwhile, the analgesic effect of the highest concentration, 0.5% ropivacaine, lasted for up to 4 h postoperatively. A pre-incision SNB also significant alleviated hemodynamic variables and reduced additional sufentanyl consumption in intraoperative period.

The incidence of moderate to severe postoperative pain developed in 22.7% of patients in the normal saline group during the first 24 hours post operation, which was similar to a previous study^17^. Nevertheless, *Gottschalk* and colleagues^18^ reported that 69% of patients undergoing craniotomies experienced moderate to severe pain during the first postoperative day. This was obviously different from the present study, potentially due to different kinds of intraoperative opioids administered: 1.1% received sufentanyl and 87% fentanyl in previous research versus induction with sufentanyl 0.2–0.3 mcg/kg in this study. Sufentanil is a potent analgesic which can produce greater and longer-lasting analgesia than equipotent doses of fentanyl. *Peter et al*.^19^ reported that the duration of analgesia with sufentanil was about twice than fentanyl. As well as we know, the statistically significant difference in scores is not equivalent to clinically important difference. As previous studies have shown, a clinically significant difference is achieved only if there is a change of assessment score that exceeds a threshold, namely minimal clinically important difference (MCID)^20,21^. A recent meta-analysis concluded that the absolutely MCID values in acute pain ranged from 8 to 40 mm^21^. In the present study, we found that SNB with 0.5% ropivacaine provided preferable analgesia which relieved postoperative pain for up to 4 h postoperatively. Compared to the normal saline group, the absolute difference value of VAS in 0.5% ropivacaine were 20 mm and 12.5 mm at 2 hours and 4 hours post operation, respectively. SNB with 0.5% ropivacaine could also decrease the incidence of moderate to severe pain up to 13.6%.

A variety of strategies are available for the prevention and treatment of postoperative pain, including narcotic analgesics, non-opioid analgesics (mostly including non-steroidal anti-inflammatory drugs), adjuvant analgesics (α_2_-agonist, NMDA receptor blocker, gabapentin, etc.), and local agents^22^. Opioids, especially morphine, are mainly used for pain control in patients receiving craniotomies. In virtue of serious effects for instance nausea and vomiting, along with respiratory depression over-sedation and miosis, opioids may lead to increased intracranial pressure and arterial carbon dioxide, interfering with postoperative physical examination, and even covering up possible devastated complications such as intracranial hemorrhage and neurological dysfunction. In a double-blind trail, *Ayoub et al*.^5^ confirmed that the implementation of the scalp block, with a mixture of 2% lidocaine and 0.5% bupivacaine after surgery, in patients undergoing a craniotomy with remifentanil-based general anesthesia provides equivalent postoperative analgesia to intravenous 0.1 mg/kg morphine given after dural closure.

Pain after craniotomy predominately derives from skin incision, muscle rupture, periosteum separation, and even dura master, rather than the parenchymal resection^23–25^. The role of the scalp block in the control of postoperative headache mainly involves blockage of the pain afferent pathways to central nervous system and enhancement of postoperative analgesic efficacy. Several studies have confirmed SNB has benefits to decrease post-operative pain and maintain the stability of hemodynamics to noxious procedures. *Jin-Young Hwang et al*. reported that SNB with 0.75% levobupivacaine at the end of surgery effectively improved recovery profiles including relieving postoperative pain, reducing patient control analgesia (PCA) consumption, and decreasing the requirement of anti-hypertension agents^26^. *Lawan Tuchinda et al*.^3^ showed that scalp block with 0.25% and 0.5% bupivacaine can blunt blood pressure and heart rate responses to noxious stimulus. *Bala et al*.^27^ observed that scalp block with 0.5% bupivacaine, added with 1:400,000 adrenaline after skin closure, decreased pain scores up to 6 hours postoperatively compared to normal saline group in patients who were received supratentorial craniotomies. In our study, SNB with 0.5% ropivacaine as postoperative analgesia had up to 4 hours of pain relief after craniotomy and was efficient in maintaining MAP and HR control. Yet, the analgesic effect of lower concentration ropivacaine (namely, 0.2% and 0.33%), only lasted 2 hours after surgery in the study. We speculate the possible reason of 0.5% ropivacaine yielding a better analgesic effect might be attributed to its higher concentration. Previous study has demonstrated that the longer the patient’s operation time, the more severe the postoperative pain^21^. In present study, stratification analysis showed only when the duration of neurosurgical procedures were more than four hours, groups R_0.2_, R_0.33_ and R_0.5_ exhibit significantly lower VAS scores than group C at postoperative 2 hours. This result revealed that scalp nerve block with ropivacaine was probably preferable to be performed for patients whose duration of surgery were long.

Additionally, in this study, SNB with either 0.2%, 0.33%, or 0.5% ropivacaine was efficient in maintaining MAP and HR stability to any noxious events (e.g. skin incision, drilling and sawing skull bone). Intraoperative HR was significant lower in 0.2% and 0.5% ropivacaine groups when compared to control group at incision drilling and sawing. 0.33% ropivacaine failed to relieve HR response to dramatic irritation, and be potentially explained by two patients’ responses (105 and 108 beats per minute) in this group with a preoperative history of sinus tachycardia. Although the baseline of HR was no statistical difference among groups (P = 0.122), this benign changes in HR (sinus tachycardia) might mask the positive effect of scalp nerve block. Induction with sufentanyl 0.2–0.3 mcg/kg probably attenuated the clinical effect of SNB to intraoperative HR variability. Besides, at different time points, MAP and HR had statistically significant changes over time. But covariance of time was also not statistically significant, which manifested that the trend of MAP and HR changes over time in every patient was similar. Several previous studies^1,3,8,28,29^ reported preference to SNB for intraoperative hemodynamics control and a few of those evaluated analgesic consumption between blockade group and control group intraoperatively. *Lee et al*.^29^ concluded that scalp block with 0.25% bupivacaine was an effective adjuvant treatment to provide hemodynamic stability and reduce the need of supplemental intravenous or volatile anesthetics. In our study, we compared analgesic consumption and observed that additional intraoperative sufentanyl requirements were dramatically lower in 0.2%, 0.33% and 0.5% ropivacaine groups than control group. Additionally, there is no difference in effect within intervention groups, as assessed by multiple comparison tests.

Opioids, regardless of the potent efficacy, were commonly accompanied with an unwanted side effect: PONV^30^, which limit its application and may contribute to postoperative pain uncontrolled in patients with craniotomy. *Zhou et al*.^17^ reported skin infiltration with 0.5% ropivacaine significantly reduced PONV at 8 hours compared to the normal saline group, in which intravenous morphine is a rescue analgesic postoperatively. However, *Lawan Tuchinda et al*.^3^ reported that scalp nerve block with 0.25%, 0.5% bupivacaine containing 1: 20,000 epinephrine did not reduce the dosage of postoperative analgesic-morphine, and did not alleviate PONV. Similarly, in our study, the incidence of PONV had no significant difference between groups. One reason explaining this finding would be that patients undergoing craniotomy were often intravenously administered corticosteroid, a potent antiemetic, aiming to relieve postoperative encephaledema in the ward. Additionally, antiemetics prophylaxis and ERAS protocol may contribute to this finding. Also, these results could be related to the use of dezocine as a rescue analgesic in this study. Dezocine, an opioid receptor agonist-antagonist, has potency similar to that of morphine, with faster onset of action than morphine and less postoperative complications than morphine^31–33^. A recent meta-analysis, however, indicated the incidence of PONV was no different following dezocine treatment compared with placebo or morphine^33^. In our study, the four groups are similar in dezocine consumption as rescue analgesic and time to first requirement of dezocine, which resembled what *Lawan Tuchinda* reported^3^. It was likely that the limited sample size of the trails also contributed to this result.

We chose ropivacaine in the study mainly because of its superior safety profile without high incidence of toxicity and a shorter duration of action with long-lasting analgesic effect. With a vasoconstrictive effect at low concentration (0.063–0.5%)^34^, ropivacaine is often not used with epinephrine^7^ which also has an unpredictable role in the cardiovascular system. The concentrations of ropivacaine in this study were chosen to be 0.2%, 0.33%, 0.5% based on the findings that scalp infiltration by 0.5% ropivacaine reduced postoperative pain received craniotomy in previous study^17^ and 0.2%, 0.33% ropivacaine are usually preferred for peripheral nerve block procedures^35–37^. In our study, pre-incision SNB with 0.2% and 0.33% ropivacaine decreased the postoperative pain for up to 2 hours, and 0.5% ropivacaine used as postoperative analgesia had longer duration for up to 4 hours after craniotomy. We speculated this phenomenon might be due to preemptive analgesia. SNB before incision may prevent the central sensitization triggered by surgery and inhibit inflammatory factors before the cascade reaction. In contrast, in a prior study, *Nguyen et al*.^38^ observed the duration of analgesia was much longer than expected with administration of 0.75% ropivacaine for a scalp block after skin closure. They explained that this long-acting effect of local agent was due to the nerve block at the end of surgery. As such, nerve blockade before surgical incision had significant effects on reduction of hemodynamic fluctuation.

Several previous studies reported^1,39^ that about 15% to 50% of patients were distressed by persistent postoperative headache after craniotomy. Although we have assessed pain in the first postoperative day, we neglected long-term follow-up to evaluate that the effect of SNB with ropivacaine on chronic postoperative pain. Furthermore, we may pay attention to the addition of new drugs such as dexmedetomidine and dexamethasone as an adjuvant to local agents in order to increase the duration of the scalp nerve block in future research.

## Materials and Methods

### Patients

We strictly followed Helsinki declaration and Chinese guidelines of Good Clinical Practice. After Institutional Ethics Committee of West China Hospital of Sichuan University approval (Ethical Committee No. 178) on 5 November 2014 and registered in the Chinese Clinical Trial Registry (registration number: ChiCTR-IPR-15006030, registration time: 26/11/2014), patients aged 18 to 60 years, who were waiting for elective craniotomy that acquired general anaesthesia were enrolled in this prospective, randomized, placebo-controlled and double-blind study from March 2015 to October 2015, with American Society of Anesthesiologists status I or II (ASA I or II) and body mass index 18 to 30 kg/m². Patients were excluded if they were unable to understand or use VAS, allergic to local anesthetics, those with Glasgow coma scale scores ˂15, and history of opioid dependence, coagulopathy, scalp infection, pregnancy and previous craniotomy. Withdrawal criteria included complications of surgery aphasia, unconsciousness, postoperative mechanical ventilation and duration of surgery longer than 6 hours.

The pre-anesthesia visit was to occur within 1 day before the scheduled surgery by one non-participating anesthetist. All patients who were screened for enrollment and met eligibility criteria, were given verbal explanations of the 10-cm visual analogue scale (VAS) scores (0 cm: no pain, 10 cm: the worst pain imaginable) for pain assessment. Every participant must be given a written informed consent.

### Randomization

The SNB was performed by one anesthetist who was blinded to the agents which has been prepared by a nurse anesthetist non-involved in the study in identical syringes. Another anesthetist who does not participate in data management and statistical analysis will generate random numbers in a 1:1:1:1 ratio. Patients were divided into one of four groups with computer-generated randomized numbers in sealed envelopes.

### Anesthesia

The anesthesia protocol and monitoring were standardized for all patients. No premedication was given. Monitoring, which consisted of electrocardiography, saturation of pulse oximetry, end-tidal carbon dioxide pressure, and invasive blood pressure measurement, was initiated by a Philips M1167A monitor (Hewlett-Packard, Boeblingen, Germany). After pre-oxygenation, anesthetic induction was performed with midazolam 0.03–0.05 mg/kg, sufentanyl 0.2–0.3 mcg/kg, propofol 1.5–2.0 mg/kg, and rocuronium 0.5–0.7 mg/kg. After tracheal intubation, propofol (4~12 mg/kg/h), remifentanil (0.1~0.2 μg/kg/min) and 0.5–1.0 minimum alveolar concentration (MAC) of sevoflurane mixed with oxygen (50%) and air (50%) was used for maintenance of anesthesia and adjusted to maintain mean arterial pressure (MAP) ranging from 60 to 80 mmHg. Ventilation was mechanically controlled to maintain an end-tidal carbon dioxide of 30–35 mmHg. Urine output were also monitored.

MAP and heart rate (HR) were measured at the following time points: baseline, three minutes before skin incision, three minutes after skin incision, drilling, sawing skull bone and skin closure. Concentrations of sevoflurane or propofol were adjusted if intraoperative blood pressure increased by more than 15 mmHg or if HR increased by more than 10 beats/minute. If the adjustment failed, an additional 0.05 μg/kg of sufentanyl was given to prevent sympathetic response.

An intramuscular injection of 5 mg dezocine was given as a postoperative rescue analgesic when patients had a VAS pain score of 4 cm or more than 4 cm.

### Scalp nerve block

The SNB was done before surgerical incision and after intubation, and included injection into unilateral supraorbital, auriculotemporal and lesser occipital nerve of the side of craniotomy, and bilateral greater occipital nerve. The block was performed by a blinded investigator who did not visit of patients and the technique was previously described by Pinosky *et al*.^8^. The anesthetic solution which consisted of 0.2% ropivacaine (group R_0.2_) (Naropin (10 mg/ml), AstraZeneca), 0.33% ropivacaine (group R_0.33_), 0.5% ropivacaine (group R_0.5_) or 0.9% normal saline (group C) is 8 ml totally and free of adrenaline.

### Outcomes

The primary measured outcome was the score of postoperative pain which was assessed at time 2, 4, 6, and 24 hours postoperatively. Secondary outcomes were intraoperative hemodynamic variables (MAP and HR) and additional sufentanyl requirements. The total consumption of dezocine during the first 24 hours after surgery and the time to first injection was calculated. The incidence of postoperative nausea and vomiting (PONV) and complications both from local anesthetic and the nerve block were also assessed.

### Statistics

Statistical analysis was performed by a statistical package program for PC-SPSS version 23.0 (Statistical Program for Social Sciences, SPSS Inc, Chicago, Illinois, USA). Demographic data and occurrence of nausea and vomiting were compared using χ² tests and analysis of variance (ANOVA), as appropriate. VAS scores, additional sufentanyl given during surgery, total consumption of dezocine was evaluated using Kruskal-Wallis test. Stratification analysis of the surgerical duration (≤4 h and >4 h) were performed to evaluate the effect of duration on postoperative pain. Hemodynamic variables and VAS scores were compared by generalized linear mixed model (GLMM) using group as fixed effect and time as random effect. Bonferroni test for multiple comparisons was used as a follow-up technique for pairwise comparison to further investigate any statistically significant findings. Time to first dezocine requirement compared using log-rank test. A value of p < 0.05 was considered statistically significant.

## Conclusion

SNB with ropivacaine before surgical incision can provide superior postoperative analgesia: blockage with 0.2% and 0.33% ropivacaine decreased postoperative pain of patients for up to 2 hours while use of 0.5% ropivacaine as postoperative analgesia provided a longer duration of action of 4 hours post operation. SNB with 0.2%, 0.33%, or 0.5% ropivacaine without epinephrine can blunt the MAP response to dramatic stimuli as incision, drilling, as well as sawing skull bone. Additionally, use of 0.2% and 0.5% ropivacaine can decrease heart rate during the incision and drilling. Although there was no advantage of scalp block with ropivacaine in the total consumption of dezocine, the time to first dezocine requirement during the first postoperative 24 hours and the incidence of PONV, SNB with ropivacaine reduced the intraoperative additional sufentanyl requirement. In conclusion, scalp block using 0.5% ropivacaine obtain preferable postoperative analgesia compared to lower concentrations (0.2% or 0.33%) in the absence of complications.

## Supplementary information

Supplementary Information.
