# Supplementary material for: Effect of Scalp Nerve Block with Ropivacaine on Postoperative Pain in Patients Undergoing Craniotomy: A Randomized, Double Blinded Study
Source: Sci Rep. 2020 Feb 13;10:2529. doi: 10.1038/s41598-020-59370-z (PMC7018808; doi:10.1038/s41598-020-59370-z)
Supplement: Supplementary file 1 — Supplementary Information. [file 41598_2020_59370_MOESM1_ESM.docx]

**Supplementary Information**

**Effect of Scalp Nerve Block with Ropivacaine on Postoperative Pain in Patients Undergoing Craniotomy: A Randomized, Double Blinded Study**

Yaoxin Yang ^1, 2*^, M.D., Mengchan Ou ^1,2*^, M.D., Hongyu Zhou ^1^, M.D., Lingcan Tan ^1^, M.D., Yajiao Hu ^1^, M.D., Yu Li ^1^, M.D., Tao Zhu ^1^, Ph.D.

**Chronological changes in hemodynamics (MAP and HR) and VAS scores by generalized linear mixed model (GLMM)**

The GLMM was based on lineal model distribution and identity link function, groups selected as fixed effect and time as random effect. We chose the model with the smallest AIC as best model. The formula of model was Y_ij_=μ_0_+ group_i_ +μ_0j_+ε_ij_. In the formula, μ_0_ represents intercept of fixed effect, group_i_ represents coefficient of different group, μ_0j_+ε_ijk_ represents intercept and coefficient of random effects .Supplementary Table 1-3 show the coefficients of fixed and random effects in MAP. Supplementary Table 4-6 show the coefficients of fixed and random effects in HR. Supplementary Table 7-9 show the coefficients of fixed and random effects in VAS.

**Supplementary** **Table 1.** **Fixed coefficients of GLMM in MAP**

| Model term | Coefficient | Std.Error | t | P value | 95% Confidence interval | |
| --- | --- | --- | --- | --- | --- | --- |
|  |  |  |  |  | **Lower** | **Upper** |
| Intercept | 82.183 | 1.618 | 50.797 | <0.0001 | 79.005 | 85.362 |
| Group R_0.2_ | -7.106 | 2.315 | -3.069 | 0.002 | -11.654 | -2.557 |
| Group R_0.33_ | -9.759 | 2.345 | -4.162 | <0.0001 | -14.365 | -5.153 |
| Group R_0.5_ | -7.805 | 2.288 | -3.412 | 0.001 | -12.301 | -3.311 |
| Group C | 0^a^ |  |  |  |  |  |

^a^ This coefficient is set to zero because it is redundant. Group R_0.2_ = 0.2% ropivacaine, Group R_0.33_ = 0.33% ropivacaine, Group R_0.5_ = 0.5% ropivacaine, Group C = normal saline.

**Supplementary Table 2. Residual effect of GLMM in MAP**

| Residual effect | Estimate | Std.Error | Z | P value | 95% Confidence interval | |
| --- | --- | --- | --- | --- | --- | --- |
|  |  |  |  |  | **Lower** | **Upper** |
| Var(T_baseline_) | 519.262 | 82.064 | 6.328 | <0.0001 | 380.946 | 85.362 |
| Var(T_before incision_) | 60.827 | 15.436 | 3.941 | <0.0001 | 36.990 | 100.024 |
| Var(T_after incision_) | 124.203 | 23.599 | 5.263 | <0.0001 | 85.587 | 180.244 |
| Var(T_drilling_) | 14.681 | 12.974 | 1.132 | 0.258 | 2.597 | 82.984 |
| Var(T_sawing skull_) | 12.621 | 12.547 | 1.006 | 0.314 | 1.798 | 88.578 |
| Var(T_skin closing_) | 61.762^a^ |  |  |  |  |  |

^a^ This coefficient is set to zero because it is redundant. T_baseline_= time points of baseline, T_before incision_ = time points of before incision, T_after incision_ = time points of after incision, T_drilling_ = time points of drilling, T_sawing skull_= time points of sawing skull, T_skin closing_= time points of skin closing

**Supplementary Table 3.** **Random effect of GLMM in MAP**

| Random effect | Estimate | Std.Error | Z | P value | 95% Confidence interval | |
| --- | --- | --- | --- | --- | --- | --- |
|  |  |  |  |  | **Lower** | **Upper** |
| Var(Intercept) | 51.136 | 9.596 | 5.329 | <0.0001 | 32.759 | 69.642 |
| Var(time) | 3.859 | 11.346 | 0.340 | 0.734 | 0.202 | 201.079 |

^a^ This coefficient is set to zero because it is redundant.

**Supplementary Table 4. Fixed coefficients of GLMM in HR**

| Model term | Coefficient | Std.Error | t | P value | 95% Confidence interval | |
| --- | --- | --- | --- | --- | --- | --- |
|  |  |  |  |  | **Lower** | **Upper** |
| Intercept | 79.193 | 2.159 | 36.685 | <0.0001 | 74.951 | 84.364 |
| Group R_0.2_ | -9.780 | 3.089 | -2.985 | 0.003 | -15.289 | -3.152 |
| Group R_0.33_ | -6.435 | 3.128 | -1.781 | 0.076 | -11.717 | 0.575 |
| Group R_0.5_ | -8.531 | 3.053 | -2.603 | 0.010 | -13.945 | -1.949 |
| Group C | 0^a^ |  |  |  |  |  |

^a^ This coefficient is set to zero because it is redundant.

**Supplementary Table 5. Reisdual effect of GLMM in HR**

| Residual effect | Estimate | Std.Error | Z | P value | 95% Confidence interval | |
| --- | --- | --- | --- | --- | --- | --- |
|  |  |  |  |  | **Lower** | **Upper** |
| Var(T_baseline_) | 112.340 | 27.982 | 4.015 | <0.0001 | 68.947 | 183.043 |
| Var(T_before incision_) | 82.406 | 24.784 | 3.325 | 0.001 | 45.704 | 148.581 |
| Var(T_after incision_) | 33.693 | 21.553 | 1.563 | 0.118 | 9.617 | 118.042 |
| Var(T_drilling_) | 73.114 | 25.345 | 2.885 | 0.004 | 37.062 | 144.237 |
| Var(T_sawing skull_) | 13.593 | 19.861 | 0.684 | 0.494 | 0.776 | 238.239 |
| Var(T_skin closing_) | 87.216^a^ |  |  |  |  |  |

^a^ This coefficient is set to zero because it is redundant. T_baseline_= time points of baseline, T_before incision_ = time points of before incision, T_after incision_ = time points of after incision, T_drilling_ = time points of drilling, T_sawing skull_= time points of sawing skull, T_skin closing_= time points of skin closing

**Supplementary Table 6. Random effect of GLMM in HR**

| Random effect | Estimate | Std.Error | Z | P value | 95% Confidence interval | |
| --- | --- | --- | --- | --- | --- | --- |
|  |  |  |  |  | **Lower** | **Upper** |
| Var(Intercept) | 91.384 | 16.683 | 5.478 | <0.0001 | 63.896 | 130.698 |
| Var(time) | 17.622 | 18.049 | 0.976 | 0.329 | 2.367 | 131.193 |

**Supplementary Table 7. Fixed coefficients of GLMM in VAS**

| Model term | Coefficient | Std.Error | t | P value | 95% Confidence interval | |
| --- | --- | --- | --- | --- | --- | --- |
|  |  |  |  |  | **Lower** | **Upper** |
| Intercept | 2.235 | 0.237 | 9.412 | <0.0001 | 1.768 | 2.702 |
| Group R_0.2_ | -0.776 | 0.340 | -2.284 | 0.023 | -0.273 | -0.108 |
| Group R_0.33_ | -0.577 | 0.344 | -1.678 | 0.094 | -0.231 | 0.100 |
| Group R_0.5_ | -0.977 | 0.336 | -2.910 | 0.04 | -0.231 | -0.317 |
| Group C | 0^a^ |  |  |  |  |  |

^a^ This coefficient is set to zero because it is redundant.

**Supplementary Table 8. Reisdual effect of GLMM in HR**

| Residual effect | Estimate | Std.Error | Z | P value | 95% Confidence interval | |
| --- | --- | --- | --- | --- | --- | --- |
|  |  |  |  |  | **Lower** | **Upper** |
| Var(T_post-2h_) | 0.420 | 0.344 | 1.222 | 0.222 | 0.084 | 2.091 |
| Var(T_post-4h_) | 0.079 | 0.321 | 0.245 | 0.807 | 0.000 | 235.117 |
| Var(T_post-6h_) | 0.237 | 0.290 | 0.815 | 0.415 | 0.021 | 2.620 |
| Var(T_post-24h_) | 1.108^a^ |  |  |  |  |  |

^a^ This coefficient is set to zero because it is redundant. T_post-2h_= time of postoperative 2h, T_post-4h_= time of postoperative 4h, T_post-6h_= time of postoperative 6h, T_post-24h_= time of postoperative 24h.

**Supplementary Table 9. Random effect of GLMM in VAS**

| Random effect | Estimate | Std.Error | Z | P value | 95% Confidence interval | |
| --- | --- | --- | --- | --- | --- | --- |
|  |  |  |  |  | **Lower** | **Upper** |
| Var(Intercept) | 1.050 | 0.201 | 5.229 | <0.0001 | 0.721 | 1.527 |
| Var(time) | 0.438 | 0.275 | 1.595 | 0.111 | 0.128 | 1.497 |

^a^ This coefficient is set to zero because it is redundant.
